# Linear behavior in Covid19 epidemic as an effect of lockdown

**DOI:** 10.1186/s13362-020-00095-z

**Published:** 2020-11-30

**Authors:** Dario Bambusi, Antonio Ponno

**Affiliations:** 1 Dipartimento di Matematica “Federigo Enriques”, Università degli Studi di Milano, Via Saldini 50, I-20133 Milano, Italy; 2 Dipartimento di Matematica “T. Levi-Civita”, Università degli Studi di Padova, Via Trieste 63, I-35121 Padova, Italy

**Keywords:** Mathematical epidemiology, Statistical models, Linear growth, COVID-19

## Abstract

We propose a mechanism explaining the approximately linear growth of Covid19 world total cases as well as the slow linear decrease of the daily new cases (and daily deaths) observed (in average) in USA and Italy. In our explanation, we regard a given population (the whole world or a single nation) as composed by many sub-clusters which, after lockdown, evolve essentially independently. The interaction is modeled by the fact that the outbreak time of the epidemic in a sub-cluster is a random variable with probability density slowly varying in time. The explanation is independent of the law according to which the epidemic evolves in the single sub cluster.

## Introduction

It is quite striking to observe the graph of the total number of cases in the world epidemic of Covid19: since the beginning of April to the half of May it is growing approximately linearly. The daily new cases of USA and Italy also exhibit a strange behavior: up to fluctuations, they decrease approximately linearly.^1^ Similarly the daily deaths of USA and Italy, but also of UK exhibit an approximately linear decrease in a slightly successive period. The linear behavior occurs after a transient in which the epidemic grows essentially in agreement with the standard laws of epidemiological models like SIS or SIR, but it is hard to explain it using such models. In the present paper we propose a mechanism in order to explain the above mentioned behaviors.

The first ingredient of our mechanism is lockdown. The situation we want to model is that of a large cluster (the world or a single nation) consisting of several sub-clusters that, due to a lockdown intervention, become almost completely disconnected from each other. One thus gets a situation with many separated sub clusters in which the epidemic evolves independently. A small interaction among the sub clusters can persist, and we model this by assuming that the time at which the epidemics outbreaks in a sub cluster is distributed at random. To take into account the fact that the evolution in each sub-cluster is characterized by different parameters we treat parameters as random variables on which the total number of cases $N_{j}$ in the *j*th cluster can depend. Then the total number of cases at a given time is the sum of many random variables, namely the quantities $N_{j}$. Assuming that the times of outbreak and the parameters describing the epidemic in a sub-cluster are independent, identically distributed random variables, it turns out that, up to a small correction, and after some transient, the expectation of the total number of cases at time *t* turns out to be proportional to the distribution function of the probability measure describing the outbreak times (the hypothesis of independence of the above mentioned random variables can be relaxed to that of weak correlation; see Remark 2.1 below). If one assumes that the probability density $\rho (\tau )$ of the outbreak times is a function of the form

1 $$ \rho (\tau )=\frac{1}{T}\varrho \biggl( \frac{\tau }{T} \biggr) , $$

with a large time *T*, then it is natural to expand in Taylor series in $\tau /T$ retaining only the first few terms. Then one can compute the expectation of the total number of cases, which (after some transient time, and with some mild assumption on *ϱ*) turns out to be close to

2 $$ c_{0}+c_{1}(t/T)+c_{2}(t/T)^{2}+O \bigl((t/T)^{3}\bigr) , $$

with suitable constants $c_{0}<0$, $c_{1}>0$, and $c_{2}<0$. Of course this is a parabola with small curvature, which is close to a line until the quadratic term becomes relevant; on such a second time scale, one observes a linear behavior of the daily new cases (i.e. the derivative of (2)). The interesting issue of estimating the time *T* and other parameters of the model from the data of the epidemics, which requires a quite careful analysis, is faced in a subsequent work.

We emphasize that, as it will be clear from the proof, our explanation predicts that the linear behavior should occur *while the epidemic is passing its pick phase* and that the behavior of the total number of cases *is independent of the specific epidemic model in each cluster*.

We conclude this section by recalling that an alternative explanation of the linear growth of the *official* cases has been proposed in [1] as a result of a limited testing capacity: the idea is that if one can test X cases per day, than he will not be able to discover more than X cases per day. However, we think that this mechanism does not explain neither the linear growth of the deaths, which is less susceptible to measurement biases, nor the large scale of the phenomenon (the world). Furthermore such an explanation does not allow to understand the linear decrease observed in the data.

Finally, we think that it would be very interesting to try to understand the origin of the probability distribution of the outbreak times through models taking into account the spatial spreading of the epidemic for example by exploiting the ideas of [2–5]. This however requires a quite detailed study of the spatial model of the epidemics and is left for future work.

In Sect. 2 we give our result. The Section is split in 3 subsections: in the first one we present our assumptions, in the second one we present an abstract theorem and in the third one we show that in the case of slowly changing probability distribution one gets the wanted linear behavior.

## Model and results

### The model

Let us consider a cluster $\mathcal {C}$ consisting of a large number *K* of disconnected sub-clusters $\mathcal {C}_{j}$, $j=1,\dots ,K$, and denote by $N_{j}(t)$ the total number of cases or deaths at time *t* in $\mathcal {C}_{j}$. For the sake of definiteness, we will simply refer to $N_{j}$ as the number of cases, without distinguishing between total cases, deaths or else. If $p_{j}$ denotes the population of $\mathcal {C}_{j}$, then $n_{j}(t):=N_{j}(t)/p_{j}$ is the fraction, or density of cases there.

In order to model the differences among the sub-clusters we assume $n_{j}$ to depend on some parameters *ω* varying in some probability space Ω. Moreover, the epidemic is supposed to start in $\mathcal {C}_{j}$ at some time $\tau _{j}$. We assume that there exists a smooth function $0\leq n(\omega ,t)\leq 1$, non decreasing with respect to *t*, s.t.

3 $$\begin{aligned}& n_{j}(t)=n(\omega _{j},t-\tau _{j}) , \end{aligned}$$

4 $$\begin{aligned}& n(\omega ,t)=0 , \quad \forall t\leq 0, \forall \omega \in \Omega . \end{aligned}$$

In particular the assumption (4) means that no history of the epidemic exists before the outbreak at $t=0$.

The number of cases in the total cluster $\mathcal {C}:=\bigcup_{j=1}^{K}\mathcal {C}_{j}$, with total population $P_{K}=\sum_{j=1}^{K}p_{j}$, is the sum $N(t):=\sum_{j=1}^{K}N_{j}(t)=\sum_{j=1}^{K}p_{j}n_{j}(t)$, and its density is the population weighted average

5 $$ \eta _{K}(t):=\frac{N(t)}{P_{K}}=\sum _{j=1}^{K}\frac{p_{j}}{P_{K}} n_{j}(t) . $$

The limit of $\eta (t)$ of $\eta _{K}(t)$ as $K\to +\infty $ is the relevant dynamical variable considered in the present work. In order to ensure its existence and to be able to compute it we make the following assumptions.

H0. $\lim_{K\to +\infty }p_{K}/P_{K}=0$, i.e. most of sub-populations do not have a macroscopic size.
H1. $\omega _{1},\dots ,\omega _{K}$ are i.i.d. random variables, distributed according to a probability measure $\mathrm {d}\mu (\omega )$ on Ω.
H2. $\tau _{1},\dots ,\tau _{K}$ are non negative i.i.d. random variables, distributed according to a probability measure $\mathrm {d}R(\tau ):=\rho (\tau )\, \mathrm {d}\tau $.

As a consequence of the hypotheses H0–H2, the density of cases in $\mathcal {C}$ defined in (5), namely

6 $$ \eta _{K}(t,\omega _{1},\ldots,\omega _{K},\tau _{1},\ldots,\tau _{K})= \sum _{j=1}^{K}\frac{p_{j}}{P_{K}} n(t-\tau _{j},\omega _{j}) , $$

becomes a weighted average of *K* mutually independent, identically distributed random variables defined, for any *t*, on the probability space $\mathbb {R}^{+}\times \Omega $ endowed by the measure $\mathrm {d}R\, \mathrm {d}\mu $. Moreover, since $n(t,\omega )$ is positive and upper bounded by definition, by the weak law of large numbers the limit

7 $$ \eta _{K}(t,\omega _{1},\ldots,\omega _{K},\tau _{1},\ldots,\tau _{K}) \stackrel{K \to +\infty }{\longrightarrow }\eta (t):= \int n(\omega ,t- \tau )\, \mathrm {d}R(\tau )\, \mathrm {d}\mu (\omega ) $$

exists in probability; see for example Theorem 1 in [6].

#### Remark 2.1

One can substitute the assumptions H1, H2 above with the assumption that the variables $\omega _{j}$ and $\tau _{j}$, and as a consequence the variables $n_{j}:=n(t-\tau _{j},\omega _{j})$, be just weakly correlated (namely, assumption (b) of Theorem 1 in [7]). This amounts to assume that the correlation between $n_{i}$ and $n_{j}$ decays sufficiently fast with the distance of the sub-clusters $\mathcal {C}_{i}$ and $\mathcal {C}_{j}$. Of course, in this case the sub-clusters are to be thought of as the nodes of a network with suitably weighted connections. A precise mathematical formulation of such a weaker assumption is also faced in a subsequent work.

### An abstract result

Since $n(t,\omega )$, as a function of *t* is a bounded non decreasing function, the limit

8 $$ n_{\infty }(\omega ):=\lim_{t\to +\infty }(t, \omega ) $$

exists point-wise and furthermore, by the dominated convergence theorem

9 $$ \lim_{t\to +\infty } \bigl\langle {n(t)} \bigr\rangle _{\Omega }= \langle {n_{\infty }} \rangle _{\Omega }, $$

where

$$ \langle {\cdot } \rangle _{\Omega }:= \int _{\Omega }(\cdot )\, \mathrm {d}\mu (\omega ) . $$

Denote by

$$ R(t):= \int _{0}^{t} \mathrm {d}R(s)= \int _{0}^{t}\rho (s)\, \mathrm {d}s $$

the distributions function of the measure d*R*, then the following theorem holds.

#### Theorem 1

*For any* $\epsilon >0$ *there exists* $T_{\epsilon }>0$ *such that for any* $t>T_{\epsilon }$ *the expected density of cases defined in* (7) *is given by*

10 $$ \eta (t)= \langle {n_{\infty }} \rangle _{\Omega }R(t-T_{\epsilon })+C_{R}(t)+\delta (t) , $$

*where*

11 $$ C_{R}(t):= \int _{0}^{T_{\epsilon }} \bigl\langle {n(s)} \bigr\rangle _{\Omega }\rho (t-s)\, \mathrm {d}s , $$

*and*

12 $$ \bigl\vert \delta (t) \bigr\vert < \epsilon . $$

#### Proof

First of all,

$$ \eta (t)= \int _{0}^{+\infty } \bigl\langle {n(t-\tau )} \bigr\rangle _{\Omega }\rho (\tau )\, \mathrm {d}\tau = \int _{0}^{t} \bigl\langle {n(s)} \bigr\rangle _{\Omega }\rho (t-s)\, \mathrm {d}s, $$

where we used Eq. (4). Now, by (9), for any $\epsilon >0$ there exists $T_{\epsilon }>0$ such that, for $t>T_{\epsilon }$ one has $\vert \langle {n_{\infty }} \rangle _{\Omega }- \langle {n(t)} \rangle _{\Omega }\vert <\epsilon $ and thus

13 $$\begin{aligned} \eta (t) =& \int _{0}^{T_{\epsilon }} \bigl\langle {n(s)} \bigr\rangle _{\Omega }\rho (t-s)\, \mathrm {d}s+ \int _{T_{\epsilon }}^{t} \bigl\langle {n(s)} \bigr\rangle _{\Omega }\rho (t-s)\, \mathrm {d}s \\ =&C_{R}(t)+ \int _{T_{\epsilon }}^{t} \langle {n_{\infty }} \rangle _{\Omega }\rho (t-s)\,\mathrm {d}s+ \int _{T_{\epsilon }}^{t} \bigl[ \bigl\langle {n(s)} \bigr\rangle _{\Omega }- \langle {n_{\infty }} \rangle _{\Omega }\bigr]\rho (t-s)\,\mathrm {d}s \\ =&C_{R}(t)+ \langle {n_{\infty }} \rangle _{\Omega }R(t-T_{\epsilon })+\delta (t) , \end{aligned}$$

where $\delta (t)$ is defined as the third integral appearing on the second line above, and clearly $|\delta (t)|<\epsilon R(t-T_{\epsilon })\leq \epsilon $. □

#### Remark 2.2

The quantity $C_{R}(t)$, defined in (11) represents the contribution of the sub clusters in which the epidemic started between $t-T_{\epsilon }$ and *t* and thus has not yet reached an end at time *t*.

### Linear behavior

We first remark that a trivial case of linear behavior is that corresponding to a uniform distribution *R*.

#### Corollary 1

*Assume that*

$$ \rho (\tau )=\frac{1}{T}\chi _{[0,T]}(\tau ) , $$

*with some* *T* *fulfilling* $T>T_{\epsilon }$, *then*, *for times* *t* *fulfilling* $T_{\epsilon }< t< T$, *one has*

14 $$ \eta (t)= \frac{ \langle {n_{\infty }} \rangle _{\Omega }}{T}(t-T_{\epsilon })+\bar{c}+ \delta (t) , $$

*with* *c̄* *the constant given by*

$$ \bar{c}:=\frac{1}{T} \int _{0}^{T_{\epsilon }} \bigl\langle {n(s)} \bigr\rangle _{\Omega }\,\mathrm {d}s . $$

#### Proof

Under the assumptions made, the quantity $C_{R}$ defined in (11) is independent of time and equal to *c̄*. □

More generally, in order to get the behavior (2), assume that the probability density of d*R* is slowly changing in time, namely it is of the form (1) with some large *T*. Then, expanding *ϱ* in Taylor series, one has (as in multiscale expansions)

15 $$ \varrho (\mathtt{t})=\varrho _{0}+\varrho _{1}\mathtt{t}+\frac{1}{2}\varrho _{2} \mathtt{t}^{2}+O\bigl(\mathtt{t}^{3}\bigr) ,\quad \mathtt{t}=t/T , $$

with $\varrho _{j}=\mathrm {d}\varrho /\mathrm {d}\mathtt{t}|_{\mathtt{t}=0}$ ($j=0,1,2 \dots $). In particular, we are interested in densities such that $\varrho _{1}<0$. We have the following

#### Corollary 2

*Assume* (1) *and* $t>T_{\epsilon }$; *then one has*

16 $$ \eta (t)=c_{0}+c_{1}(t/T)+c_{2}(t/T)^{2}+O \bigl((t/T)^{3}\bigr)+\delta (t) , $$

*where*, *in terms of the positive small parameter*

17 $$ \lambda :=\frac{T_{\epsilon }}{T} \biggl( \langle {n_{\infty }} \rangle _{\Omega }-\frac{1}{T_{\epsilon }} \int _{0}^{T_{\epsilon }} \bigl\langle {n(s)} \bigr\rangle _{\Omega }\,\mathrm {d}s \biggr) , $$

*the coefficients of the expansion* (16) *read*

18 $$\begin{aligned}& c_{0}:=-\varrho _{0}\lambda +O\bigl(\lambda ^{2}\bigr) ; \end{aligned}$$

19 $$\begin{aligned}& c_{1}:=\varrho _{0} \langle {n_{\infty }} \rangle _{\Omega }-\varrho _{1}\lambda +O\bigl(\lambda ^{2}\bigr) ; \end{aligned}$$

20 $$\begin{aligned}& c_{2}:=\frac{1}{2}\varrho _{1} \langle {n_{\infty }} \rangle _{\Omega }- \frac{1}{2}\varrho _{2}\lambda +O\bigl(\lambda ^{2}\bigr) , \end{aligned}$$

*whereas* $|\delta |<\epsilon $ *as in Theorem *1.

#### Proof

One just computes *R* and $C_{R}$ for the considered case, by plugging the expansion (15) into both of them and collecting the terms proportional to the same power of $t/T$. □

Concerning the signs of the coefficients $c_{0}$, $c_{1}$ and $c_{2}$, we observe that $\varrho _{0}>0$ and $\lambda >0$ is small, which ensures $c_{0}<0$ and $c_{1}>0$. On the other hand, in order to ensure $c_{2}<0$ one needs $\varrho _{1}<0$, as pointed out above.

## Data Availability

We looked at the following web sites https://www.worldometers.info/coronavirus/ and https://coronavirus.jhu.edu/map.html.

## Footnotes

1 USA between half of April and the last part of May, Italy between the end of March and the beginning of May.

## Abbreviations

i.i.d.: Independent and identically distributed
